# A qualitative exploration of ultra-processed foods consumption and eating out behaviours in an Indonesian urban food environment

**DOI:** 10.1177/02601060221133897

**Published:** 2022-11-04

**Authors:** David Colozza

**Affiliations:** 1 Department of Global Health and Social Medicine, 4616 King's College London, London, UK; 2 Department of Geography, 4616 King's College London, London, UK; 3 Department of Geography, 37580 National University of Singapore, Singapore

**Keywords:** behavioural research, food and nutrition, eating behaviour, healthy diet, obesity, qualitative research

## Abstract

**Background:** Increased consumption of ultra-processed foods and drinks high in unhealthy fats, salt and sugar is a major driver behind rising non-communicable disease rates in Asia-Pacific. Urban residence is considered a risk factor for increased consumption of these products; yet, evidence on consumption behaviours and drivers from urban populations in the region remains limited. **Aim:** To understand perceptions and drivers of unhealthy fats, salt and sugar foods and drinks consumption and eating out behaviours in Yogyakarta city, Indonesia. **Methods:** In-depth qualitative data were collected through open-ended interviews and prolonged interactions in the local food environment, from a purposeful sample (*N* = 45) equally distributed across three urban communities. Data were analysed according to the principles of content analysis and following an iterative approach. **Results:** Despite showing high nutritional health awareness, respondents and their household members consumed ultra-processed foods high in unhealthy fats, salt and sugar regularly. Home consumption of these products was based primarily on economic considerations and convenience, but also related to attending requests from other family members, individual preferences and tastes, and social functions. Similarly, despite a reported preference for home-cooked traditional foods, several participants or their family members would frequently eat ready-made meals away from home, due to conflicting school or work commitments. **Discussion:** Results suggest that public health interventions focused on nutrition education among Indonesian communities should be coupled with measures addressing urban food environment characteristics that promote the consumption of unhealthy diets, be tailored to specific age groups, and leverage traditional food cultures.

## Introduction

Non-communicable diseases (NCDs) are the leading cause of premature death, responsible for about 70% of deaths worldwide (WHO, 2020). In turn, six out of 11 main risk factors for NCDs are related to diet (GLOPAN, 2017). Among diet-related risk factors are overweight and obesity, whose prevalence has grown steadily in Asia-Pacific over the past decades. The region has currently the largest absolute number of overweight and obes people globally (FAO et al., 2021).

Similarly to other low and middle-income countries (LMICs), Indonesia has experienced shifting nutritional health challenges over the past decades. Undernutrition issues, albeit declining, still persist. As of 2018, 9.3% of the adult population suffered from undernutrition and 30.8% of children under 5 years of age from stunting (Ministry of Health - RI, 2018). Concurrently, health issues linked with overnutrition have also been growing. Between 2010 and 2019, high body mass index (BMI) was the fastest-growing mortality risk factor in the country (GBD 2019 Risk Factors Collaborators, 2020) and as of 2018, 13.6% and 21.8% of Indonesian adults lived respectively with overweight and obesity (Ministry of Health - RI, 2018).

Increased consumption of foods and drinks high in unhealthy fats, salt and sugar (HFSS), including commercially produced ultra-processed foods (UPFs), savoury and sweet snacks, sugary beverages, and ready-made meals, is a key driver of rising overweight and obesity rates in LMICs (Popkin et al., 2020). Growing consumption of these products has in turn been linked with their increased availability, desirability and affordability in global food systems, resulting from free trade agreements, marketing campaigns, and the rapid penetration of modern food retailers in LMICs (Baker and Friel, 2014; Popkin et al., 2020). In parallel, socio-economic shifts across LMICs have increasingly encouraged a preference for convenience. For example, higher incomes and reduced time available to cook due to growing participation in the job market have favoured more frequent consumption of ready-made meals, UPFs and HFSS foods, at home or outside (Popkin et al., 2012).

 Urban residence is thought to amplify these shifts, as cities generally experience faster socio-economic change compared to rural areas, and offer higher access to HFSS foods and to eating-out opportunities (Leeder, 2010). Yet, evidence on the links between urban residence and diets shows mixed patterns of association (Caspi et al., 2012). In addition, available studies from LMICs have frequently focused on quantifying how structural features of the urban food environment, such as food outlet availability and access, affect diets. While the value of qualitative approaches to health and nutrition research has been widely documented (Ottrey et al., 2018; Panter-Brick and Eggerman, 2018), in-depth qualitative accounts of individual-level drivers of food consumption in Asia-Pacific, and in Indonesia specifically, remain limited (Farrell et al., 2021; Turner et al., 2020).

In this context, this article investigates UPFs and HFSS foods consumption and eating out behaviours in three urban food environments in Yogyakarta, Indonesia. Specifically, the article seeks first to elucidate the drivers of HFSS food consumption among study participants, and second, to understand their attitudes and habits related to eating outside the home, including main motives and choice of food outlet. The study adds novel evidence based on in-depth qualitative data on individual-level perceptions – a key influence on nutritional behaviours alongside that of food environments and broader socio-economic factors (Swinburn et al., 2011). In turn, a better understanding of individual perceptions and behaviours can help identify those factors that hinder healthy eating, and inform the design of more effective public health interventions.

## Methods

Data collection took place between September 2018 and April 2019, as part of a larger study on urban health and nutritional behaviours. The sample includes 45 participants across three areas of different socio-economic background: Terban (low-income), Cokrodiningratan (middle-income) and Sagan (high-income).^1^ The decision to focus on communities of different socio-economic makeup was made in recognition of the increased burden from overweight/obesity and NCDs among lower-income households, in LMICs and Indonesia specifically (Popkin et al., 2020), and to understand whether any major difference in HFSS consumption behaviours existed in the sample depending on income levels. The study aimed to gather in-depth knowledge on individual behaviours and perceptions, and thus prioritised frequent and prolonged interaction with study participants over large-scale data collection. One main criterion guided sample selection: whether the respondent was responsible for household food decision-making (e.g. food purchase and preparation). Respondent selection was first guided by conversations with local community leaders and subsequently followed a snowballing approach, with respondents suggesting additional interviewees in the area. Table 1 summarises the sample descriptive statistics.

**Table 1.** Descriptive statistics of the study sample. Figures are percentages unless otherwhise stated.

	Overall	Cokrodiningratan	Sagan	Terban
Female	93	93	93	93
Age (average)	47.7	49.8	45.2	48.2
Ethnic Javanese	93	.93	87	100
Muslim	89	100	73	93
Household size (average)	4.7	4.50	5.40	4.20
In charge of cooking for the household	93	93	93	
Originally from Yogyakarta	47	33	60	60
If not originally from Yogyakarta, migrated from a rural area	75	100	50	83
Employed	71	87	67	60
Monthly income, IDR 1 mil. (USD equivalent)				
0–0.5 (US$0–36)	13	20	0	.20
0.5–1 (US$36–72)	31	27	27	40
1–1.5 (US$72–108)	18	20	13	20
1.5–2 (US$108–143)	13	13	20	7
> 2 (> US$143)	24	20	40	13
Education				
No formal education	7	13	0	7
Primary school	11	20	0	13
Middle school	18	7	13	33
High school	44	53	33	47
University	20	7	53	0

Data were collected through in-person interviews by the principal researcher (at the time, a PhD researcher) and two undergraduate research assistants, who were already experienced in qualitative interviewing and received additional training before the start of the study. A semi-structured interview guide was developed, translated, and reviewed after pilot testing (Appendix 1). Information was collected in Indonesian, recorded, transcribed verbatim and translated into English for analysis. Participants were interviewed at home, either alone or in the presence of other members of the family. They were all briefed on the research aims and signed informed consent forms, of which they retained a copy. The study received ethical clearance from the Research Ethics Office, King's College London (Research Ethics Number: MR/17/18-244) and the Ministry of Technology, Research and Higher Education of the Republic of Indonesia (Approval Number: 2948/FRP/E5/Dit.KI/IX/2018).

Interviews (lasting between 35 min and 1.5 h) revolved around open-ended questions. These first sought to understand participants’ perceptions and consumption of HFSS foods, including commercially produced UPFs, and local savoury and sweet snacks and drinks. Secondly, questions prompted participants to discuss their eating out behaviours, particularly whether they (or someone in the family) would often eat outside and, if yes, at what outlets and on which occasions. The design of the research instruments and the subsequent analysis of data collected were informed by general theoretical frameworks that link distal and proximate socio-economic and environmental factors with individual-level nutritional health (Lang and Rayner, 2010; Swinburn et al., 1999, 2011), as well as by available literature on (urban) food environments (Downs et al., 2020; Thompson et al., 2013; Turner et al., 2020).

Interview responses were analysed based on the verbatim Indonesian text and the corresponding translation in parallel, to ensure the latter appropriately captured the discussions with participants. This approach, together with the specific time allocated prior to the start of data collection to discuss and define key terms used in the interview guides (e.g. UPFs, ‘traditional’ snacks, ready-made meals), minimised the risk of misinterpretation of questions by study participants, and of findings during the analyses. Following the principles of content analysis (Shannon and Hsieh, 2005) results were first grouped into broad themes by the principal researcher. Following the iterative procedure outlined by Tracy (2013), themes were further refined based on findings emerging from this first round of broad-brush coding, on triangulation with field notes taken before, during and after data collection, on discussions within the research team and with other local researchers, and on local evidence available in the literature. The prolonged exposure and access to study areas (roughly 7 months in total, between 2018 and 2019) also made it possible to clarify specific questions related to the food environment in the three areas that emerged during the analysis of findings, by following up directly with study participants and community leaders. Data saturation was observed in all three areas, with no additional findings emerging after approximately two-thirds of the planned interviews (or 10 out of 15) were completed. This article was prepared following the COREQ guidelines for qualitative research reporting (Tong et al., 2007). NVivo 12 (QSR International Pty Ltd) was used for data management and analysis.

## Results

Study results are presented across the three main focus themes of the study (drivers of HFSS foods and beverages consumption, eating out motives, and drivers of food outlet choice), with key sub-themes discussed and presented at the beginning of each section. Table 2 summarises themes and sub-themes and includes a short description of each.

**Table 2.** Summary of themes and sub-themes covered by the study.

Theme, sub-themes	Description
**1. *HFSS F&B consumption behaviours***	Respondents’ behaviours related to the purchase and consumption of HFSS foods and beverages, and main motives that influenced their decision to consume these products.
1a. Convenience	Purchasing HFSS foods as a time-saving strategy.
1b. Family preferences	Purchase and/or consumption on other family members’ request.
1c. Situational consumption	Occasional consumption linked with particular situations.
1d*.* Health awareness	Health concerns as a moderating factor of HFSS foods consumption.
**2. *Eating out (drivers)***	Main factors that drove participants to purchase and consume ready-made meals outside their homes.
2a. Social functions	Eating out for recreation or in connection with specific events.
2b. Necessity	Eating out as a necessity to accommodate routine commitments.
**3. *Eating out* (*outlet choice*)**	Main factors that influenced respondents’ choice of food outlet when eating outside their homes.
3a. Economic considerations	Consideration for food prices at different outlets.
3b. Health concerns	Health concerns related to nutrition and food safety

### Drivers of HFSS foods and beverages consumption

Respondents consistently reported purchasing HFSS foods, including commercially-produced UPFs such as instant noodles, packaged and instant sugary drinks, canned meats and fish products. Consumption of these foods by study participants or their family members was regular, but varied substantially in frequency, from 1 to 2 times per week to once a month. Four themes emerged when discussing drivers of HFSS foods purchase and consumption: convenience, family preferences, situational consumption and health awareness – with the first two most frequently brought up by respondents.

Convenience was mentioned as a key driver by many respondents, who reported stocking up on HFSS foods (particularly instant noodles) as an easy back-up meal option for when they did not feel like cooking or ran out of other foods:

I rarely eat [instant noodles]. Maybe once per month, if I don't have any food at home and don't want to go buy it, then I’ll just cook Indomie [local instant noodles brand]’. (Female, 59, low-income (2), Terban)^2^

Yes, I buy instant noodles. But it's not an everyday food, I stock up on them and if we’re out of food in the evening, I will cook them. (Female, 33, high-income (5), Cokrodiningratan)

Similarly, attending to other family members’ requests and preferences for HFSS foods was frequently highlighted as a key driver of consumption. Over one-third of the respondents reported in fact rarely consuming UPFs and HFSS foods, but still purchasing them for their children or grandchildren:

I might eat some [packaged foods] like once a month … or not even that often. But my children yes, they eat them frequently, every week. (Female, 58, middle-income (3), Sagan)

No, I don't eat instant noodles, but my children and husband do, once a week. (Female, 34, high-income (4), Sagan)

A third theme that emerged when discussing drivers of consumption was situational consumption, with some respondents reporting eating HFSS foods only on specific occasions, for example when receiving them as a gift, or ‘craving’ their taste and feel in a particular situation:

Yes, I eat packaged foods sometimes, usually when someone gives them to me, for example at community events. It would be a waste if I didn't eat them. (Female, 64, low-income (1), Cokrodiningratan)

I eat instant noodles perhaps once a week . I try to avoid eating them … but during the rainy season hot noodles taste so good! (Female, 54, high-income (4), Sagan)

Finally, health concerns were also mentioned by some participants as having a key influence on their HFSS foods purchase and consumption. While most respondents reported regular purchases and consumption of these products (albeit, as noted, to different degrees of frequency), many were also highly aware of their negative health impacts, and tried to limit their or their family members’ consumption on the basis of these concerns:

I used to eat [instant noodles] often, but now not anymore, maybe once a week. They say it's not good for your health. (Female, 48, middle-income (3), Terban)

I buy [packaged snacks] for my kids, but I limit consumption to once a week … the youngest in particular is sensitive to those foods, once I had to take him to the hospital because he ate too much. That's why I need to check what I buy now. (Female, 42, high-income (5), Cokrodiningratan)

### Eating out motives

Most respondents reported a preference for eating home-cooked food over consuming ready-made meals at home or eating out. Two themes emerged most frequently to explain the main motives that drove respondents to eat ready-made meals outside their homes: social functions and necessity. For many, eating out was considered a recreational activity, done sparingly and in connection with specific social functions and occasions: family outings on weekends, birthdays and holidays, casual gatherings with friends and colleagues or participation in specific social events, such as religious and community functions:

No, we rarely [eat out], only if there is a special event, like a birthday. (Female, 52, middle-income (3), Cokrodiningratan)

I rarely [eat out]; only for the elders’ group events, if they meet, then I will eat out with them. (Female, 56, low-income (29), Cokrodiningratan)

Despite this stated preference for home-cooked foods, many respondents reported that, out of necessity, they or other family members would frequently consume ready-made meals from food vendors outside the home. In most cases, this was because everyday commitments kept those who were employed or attending school outside during meal times, with their workplace or school too distant for them to return home for meals:

My eldest son eats outside every day. Because of the class schedule in college, he doesn't have time to come back home. (Female, 45, high-income (5), Terban)

My husband only eats at home on his day off. He works outside the city, he leaves in the morning and comes back at maghrib time [around 6 PM] so he must eat lunch outside. (Female, 38, high-income (5), Cokrodiningratan)

### Drivers of outlet choice

When eating out (for leisure, or due to necessity), two main themes emerged as driving the frequency and choice of food outlets. The first related to economic considerations. All respondents but one perceived eating out as more expensive than cooking at home and, consequently, reported doing it infrequently. In particular, eating at fast food-style outlets (both international and local) was perceived as more expensive than purchasing prepared traditional meals at a *warung* (small open-air or indoor shop selling a variety of items, including raw produce and/or cooked foods) or at the local market. Most respondents who occasionally ate outside reported very infrequent patronage of Western-style fast food franchises, preferring instead traditional local dishes:

I rarely buy food outside … maybe once a month I buy prepared food at the market. I look for traditional dishes, such as *gudangan*, *pecel* … the prices [at local fast food franchises] are not affordable. (Male, 58, low income (2), Sagan)

No, I don't like Mc Donald's or that kind of foods. When I go to Galeria mall … there is a fried noodles shop that I like. So, sometimes I stop by and eat there (…) but rarely, not even once a month. I don't have money for that. (Female, 67, high income (5), Terban)

The second theme related instead to health concerns, either in terms of individual nutritional health or food safety of street vendors, including in relation to facilities, cooking methods and ingredients used. These considerations limited respondents’ overall consumption of food purchased outside and guided their choice of food outlet:

Yes, I buy ready-made meals outside, maybe twice a week. I like to buy from a stall near UNY [Yogyakarta State University], it's my children's favourite. But I only do it sometimes, they use too much oil there, and they reuse the same oil many times. (Female, 45, high income (5), Terban)

Cooking meals at home is cheaper, and also more hygienic. We don't know where they wash [the vegetables] if we buy food outside. (Female, 48, middle-income (3), Terban)

I avoid street food vendors, I prefer to buy from places where they have flowing water. (Female, 35, high-income (5), Sagan)

Both my children buy chicken noodles, meatballs, and bread outside and eat it at home. Not every day, but often … but I don't, the doctor says my cholesterol is too high. (Female, 71, low income (2), Sagan)

## Discussion

Overall, study participants unanimously recognised UPFs and HFSS foods as unhealthy and reported limiting their consumption on the basis of health concerns, whether purchasing them for home consumption or when eating ready-made meals outside the home. Unlike what was observed by Rachmi et al. (2018) in other provinces within Java, there was no difference in attitude towards the consumption of UPFs between respondents of higher and lower income. Overall, respondents had a good degree of understanding of general nutritional health messaging, and particularly of the need to consume HFSS/UPFs sparingly and prefer instead fresh, home-cooked meals based on locally available nutritious foods. This finding can be at least in part explained by the extensive nutrition education efforts made over the past decades by the Indonesian government. Several participants explicitly recited the slogan ‘*empat sehat, lima sempurna*’ (‘four are healthy, five are perfect’) during the interviews – a reference to government guidelines issued in the 1950s in the context of undernutrition and micronutrient deficiency prevention efforts, and centred on the principle of dietary diversity (Colozza, 2021; Rachmi et al., 2017a). An additional explanatory factor in this sense is the longstanding tradition of nutrition education and counselling services delivery through the extensive network of community health facilities in Indonesia, which reflects the high level of decentralisation in the country (WHO, 2017b). It is worth noting however that these services are still largely focused on preventing undernutrition issues; while some examples of local initiatives geared towards overnutrition and NCDs prevention are emerging, these remain sparse and scattered (Burnet Institute, 2021; WHO, 2017b) and will likely have to be strengthened and scaled-up to respond to the increasing challenges with these conditions in the country. Participants’ perceptions and understanding of dietary health are discussed in more depth in an earlier publication deriving from this study (Colozza, 2021).

Despite the high and relatively nuanced understanding of dietary health, a certain degree of disconnect was observed between nutritional knowledge, perceptions and attitudes towards UPFs and HFSS foods, and their reported consumption within the household included in the study. Many respondents reported in fact that they or their immediate family members would consistently purchase and consume HFSS foods and beverages at home. While this was done at varying degrees of frequency, many also reported frequent consumption of ready-made meals outside the home, in line with observations that these are a significant contributor to the high-intake of HFSS foods and beverages in the country (Andarwulan et al., 2021) and across the region more broadly (Van Esterik, 2008). This disconnect between ‘declarative’ and ‘procedural’ nutritional knowledge – that is, respectively, the ability to correctly recount nutritional facts and to put them into practice in everyday life (Mete et al., 2019) – has been observed frequently in studies of nutritional behaviours, and many explanations have been proposed for it. In their review of qualitative studies on healthy eating perceptions, Bisogni et al. (2012) highlight several inter-related socio-economic and environmental factors that interact to influence nutritional behaviours. These factors include satisfying the dietary preferences of different household members, attending to social or religious functions, considerations for resources available – money, but also time and cooking skills – and physical features of the local food environment, for instance, availability and access to healthy food options. In other words, nutritional behaviours result from the balancing of competing priorities; in this sense, nutritional knowledge and health considerations are important, but so are individual taste, convenience and tending to social relationships.

All these factors were observed to a varying degree in the study. The reasons most often cited for consuming UPFs and HFSS foods were in fact request and preference of other family members, particularly children, time constraints to prepare food from scratch, attendance at social events or special occasions such as birthdays, or simply ‘cravings’ for such foods. These observations are in line with those macro-scale socio-economic shifts identified by the global nutrition transition literature as key drivers of growing HFSS foods and beverages consumption over the past few decades – for example, increased participation in the job market and reduced time available to home-cook meals, increased disposable income among a rising middle class in LMICs, and the overall increased availability and affordability of hyper-palatable UPFs in the global food supply (Monteiro et al., 2013; Popkin et al., 2012, 2020). Findings also align with existing localised qualitative evidence from food environments in Indonesia and other LMICs in Asia-Pacific (Colozza, 2021; Downs et al., 2019; Turner et al., 2020), LMICs in other world regions (Crush and Battersby, 2016; Davies et al., 2017; Menezes et al., 2018; Pereira et al., 2015) and high-income countries (Caspi et al., 2012; Pitt et al., 2017). In this sense, the observed similarities in HFSS/UPFs consumption and eating out behaviours among consumers of all income groups suggest that affordability of these products, in Indonesia as in other LMICs, is a major driver behind their consumption across all population groups (Monteiro et al., 2013), and aligns with recent findings suggesting that the fastest rise in prevalence of overweight and obesity in Indonesia has occurred among low-income households (Popkin et al., 2020).

A similar disconnect between reported perceptions and consumption behaviours was also observed for ready-made meals consumed outside the home. Study participants in Yogyakarta overall considered eating out an expensive option reserved for specific occasions, and reported a general preference for consuming home-cooked foods. Many respondents indicated in fact that they (or their family members) would often eat ready-made meals, either outside or at home, primarily due to conflicting work or school commitments that would not allow them to return home at meal times. These findings align with those of others studying drivers of eating out behaviours in the region – for example, Naidoo et al. (2017) found that in Singapore, the high availability and affordability of ready-made meals and the convenience these offered resulted in a high frequency of consumption, particularly among employed respondents. Significantly, while growing numbers of international and local fast food franchises in Southeast Asia are often interpreted as suggesting changing preference towards this food type (Pingali, 2006), most respondents who occasionally ate outside reported preferring traditional local dishes and street foods and snacks sold by local vendor types over Western-style fast food, and rarely (if at all) eating at fast food franchises. The observed retention of preference for traditional foods amidst rapid socio-economic change aligns with existing observations from Indonesia and Southeast Asia (Colozza, 2021; Colozza and Avendano, 2019; Lipoeto et al., 2001, 2004, 2013; Sukenti et al., 2016) and LMICs in other regions (O’Neill, 2015; Paddock, 2017) and suggests that local outlets and street food vendors could be a promising entry point for public health interventions aimed at reducing HFSS foods and beverages intake (Andarwulan et al., 2021).

In economic terms, it is possible that the specific context of Yogyakarta influenced eating-out behaviours. Several respondents mentioned that prices for raw foods in the city were lower compared to other provinces both within Java and country-wide. This in turn suggests that, in Yogyakarta, the cost of eating out as opposed to purchasing fresh produce to cook at home might be higher or similar, depending on the outlet. A second factor that is likely to have influenced respondents’ eating out behaviour is age, which emerged from interviews as a recurrent theme influencing dietary habits. It was mostly younger members of the family who were reportedly more eager to try foods that are new, ‘trendy’ and not usually cooked at home (including fast foods), more frequently disliked home-cooked foods, socialised with friends by meeting outside for meals, and more frequently ate out during lunch breaks at school. Similarly, and as observed by others studying food choices in Java (Rachmi et al., 2017a; Rachmi et al., 2018), respondents reported that often they would eat outside, or buy cooked food to eat at home on their children's wishes. Research looking separately at eating out behaviours specific to older and younger people (and, more broadly at their nutritional behaviours) may thus come to different conclusions.

The study has two main limitations. First, as expected from a qualitative study, findings are based on a purposive sample and derived from specific geography; as such, they cannot be used to infer conclusions on other urban food environments in Yogyakarta or elsewhere in Indonesia. Nevertheless, based on extensive in-country experience, observations, and discussions within the team and other local researchers, as well as on the inclusion of respondents of different socio-economic status, it is likely that at least some aspects of the food environment and observed consumption behaviours can reflect those of urban residents in other mid- and large-sized Indonesian cities. Second, as with all research focused on understanding food-related decision-making through self-reported measures, it is possible that information provided by respondents does not accurately reflect their actual behaviours – for example, in terms of HFSS foods and beverages consumption, patronage of fast food outlets, or reliance on ready-made meals. Yet, as the scope of the study was to understand individual perceptions and behaviours rather than quantifying exactly nutritional intake or habitual dietary patterns, this limitation is thought not to impact the validity of results presented to a significant extent.

This study focused on understanding perceptions and attitudes towards UPFs and HFSS foods consumption and eating out – two key drivers of increasing rates of overweight and obesity and NCDs in LMICs and Indonesia specifically. Regardless of income levels, participants unanimously showed high nutritional health awareness, which reflected in purposeful attempts to restrict their and their families’ consumption of unhealthy UPFs and HFSS foods, and a general preference for home-cooked traditional meals over ready-made Western-style fast food. The observed disconnect between individual knowledge of dietary health and reported behaviours suggests that interventions focused exclusively on increasing awareness of nutritional health may not be an effective tool if employed in isolation. Based on study findings, efforts focused on nutrition and health education should be accompanied by interventions seeking to improve local food environments by enabling access to healthy foods and reducing exposure to, and consumption of HFSS foods and beverages. These include, at the local scale, regulations and interventions focused on increasing access to healthy foods in public places such as schools and offices and, at national-scale, policies aligned with WHO's ‘best buys’ for NCD preventions, which can tackle the systemic drivers of overweight and obesity, including effective front-of-pack-labelling schemes, taxation on UPFs such as sugar-sweetened beverages, and regulations on their marketing, particularly to children (WHO, 2017a). In addition, considering the different reported attitudes towards the consumption of UPFs, HFSS foods and ready-made meals across age groups, interventions targeting the improvement of nutritional health knowledge, particularly as it relates to NCD risk, should also target younger age groups, such as school-age children and adolescents. Finally, the reported retention of preference for local traditional dishes suggests a possibly effective entry point for public health strategies that seek to promote nutritional health by leveraging traditional healthy food cultures.

## Notes

1. The three areas are named after their respective administrative district. Each area was heterogenous in terms of income levels. The classification in low, middle and high-income was made for the purpose of this study based on participant demographics, consultation with local residents and authorities, and visual observations during data collection (e.g. housing type and conditions, sanitation facilities available).

2. Participant information is reported as: gender, age, income level (1–5, lowest to highest) and study area.

## Appendix 1. Interview guide

**Table:**

***#***	***Background information***						
**01**	Kelurahan/RT/RW						
**02**	Location coordinates						
**03**	Name of respondent (Nama responden)						
**04**	Household ID						
**05**	Gender (Jenis Kelamin)						
**06**	Age (Umur)						
**07**	Ethnicity (Suku)						
**08**	Religion (Agama)						
**09**	Number and age of family members (*Jumlah Keluarga dan komposisi*)	Age (Umur):	0–5:	5– 15:	15–55:	> 55:	Total:
**10**	Did you move to this area from somewhere else? (*Apakah bapak/ibu asli ditinggal disini atau pindahan dari mana?*) Y/N	**10a.** If yes, was it from outside Jogya? (*Jika Iya, apakah berasal dari luar Jogja?*)			Y/N	**10b.** If Yes, was it from a rural area? (*Jika iya, apakah dari desa?*)	Y/N
		**10c.** Name of the area (*nama daerah)*			** **		
***#***	***Socio-economic information* (*Informasi berkaitan Sosial-Ekonomi*)**						
**11**	Is respondent usually the main person in charge of activities related to food (e.g. buying, cooking)? (*apakah bapak/ibu adalah orang utama yang bertanggung jawab/mengola aktivitas berhubungan dengan makanan*) (*Contoh membli, memasak*)	Y/N	**11a.** If no, who is usually in charge of this? (*jika bukan, siapa yang bertanggung jawab/yang mengelola*?				
**12**	Are there other people in the HH who take part in these tasks? Who? (*Adakah orang lain didalam rumah tangga yang mengambil alih/,mengurus pekerjaan tersebut? Siapa kah orang tersebut?*)	Y/N	**12a.** If yes, who? (*jika iya, siapa?*)				
**13**	Do you have a job? (*apakah bapak/ibu bekerja?*)	Y/N	**13a.** If yes, what is your job? (*Jika iya, apa pekerjaan bapak/ibu*)				
**14**	You household's monthly earnings are more or less between … (*Berapa pendapatan di keluarga bapak/ibu dalam satu bulan?*)	0–500,000	500,000–1 juta	1–1.5 juta	1.5–2 juta	> 2 juta	
**15**	What is the highest education grade that you completed? For example, no education, SD, SMA, University (*pendidikan terkakhir bapak/ibu?*)						
**16**	Do the children in the family (if any) attend school? What grade? (*apakah bapak memilki anak? Sekarang bersekolah dimana dan kelas berapa*?)						
** **	***Food consumption, choices and sources in the local environment***						
#	Part 1. Food consumption. 24-h dietary recall						
**17**	*Question: ‘Please describe the foods (meals and snacks) that you ate or drank yesterday during the day and night, whether at home or outside the home. Start with the first food or drink eaten in the morning’.* *Pertanyaan : Mohon bapak/ibu sebutkan dan jelaskan makanan (baik makan berat maupun hanya makan camilan) yang bapak/ibu makan dan minum kemarin dari pagi hingga malam, baik di rumah maupun diluar rumah. Mulai dari makanan pertama kali yang dimakan /minuman pada pagi hari?*						
	
Time of day: (*waktu*)	Name of food/drink (*Nama Makanan/minuman*)	Place eaten (home or away) (*Tempat makan?*)		Where did you buy it? (*dimana bapak/ibu membeli makanan/minuman tesebut?*)		Description: ingredients, cooking method* Deskripsi : bahan-bahan, cara memasak	
**#**	Part 2. Food sources and choices. (Sumber makanan dan pilihan makanan)						
**18**	Could you tell us about: *(ceritakan/jelaskan kepada kami berkaitan)* - the places (e.g. shops, markets, street vendors) where you buy foods for your family; (*tempat membeli makanan contoh warung, pasar, pedangan asongan, pedagang kaki lima*) - the foods you usually buy in each; (masing-masing, makanan yang selalu bapak/ibu beli) - and the reason for choosing them? (*dan, alasan memilih tersebut?*)				**18a.** Name/type of shop(*Nama/tipe tempat beli makan)*: **18b.** Location(*lokasi*): **18c**. Transportation(*transportasi*): **18d.** Foods bought (*makanan yang dibeli*): **18e.** Reason for choosing (*Alasan membeli*):		
**19**	Do other people in the family regularly eat, or buy food somewhere else? (*apakah ada salah satu anggota kelaurga yang biasa makan dan membeli makan diluar?*) Y/N				**19a.** If yes, can you tell us more about why, how often, what do they eat? (*Jika iya, dapat kah bapak/ibu ceritakan mengapa, dan seberapa sering mereka makan di luar*)		
**20**	We would like to show you this map of your area, where we have noted down the food shops that are close to you. (*Kami akan menunjukan kepada bapak/ibu peta, dimana kami sudah memberi tanda tempat pejual makan yang dekat dengan bapak/ibu*)			Is there any shop that is not on the map, where you or others in the family shop or eat often? (*apakah masih ada tempat penjual makanan yang tidak termasuk didalam peta,dimana bapak/ibu atau anggota keluarga yang lain membeli makan atau makan?* *Y/N*		**20a.** If yes, can you tell us what shop it is, and where it is on the map? (*Jika iya, dapatkah kamu menceritkan tempat penjual makanan tersebut, kira-kira dimana jika dilihat dari peta*)	
**21**	Could you tell us a bit about a recent trip to purchase food, for example, to the market, supermarket or other places? (*dapatkah bapak/ibu menceritakan perjalanan atau proses bapak/ibu untuk membeli makan, sebagai contoh di pasar, supermarket atau tempat lain? Perjalanan paling terkahir bapak/ibu membeli makanan)* Please tell us about how do usually go to this place, how much time you normally spend there and how you decide what food to buy when there (e.g. a pre-prepared list, or see what is on sale or depending on prices etc.) *(mohon bapak/ibu ceritakan mengenai seberapa sering pergi ke tempat tersebut? Biasanya berapa lama menghabiskan waktu untuk membeli dan pergi ke tempat tersebut? Bagaimana bapak/ibu memutuskan untuk membeli makanan /minuman tersebut (sudah menyiapkan daftar yang akan dibeli, melihat apa yang sedang dijual, atau tergantung pada harga))*						
**22**	Do you often eat outside of your home, or buy prepared foods outside (e.g. at a warung or fast food shop) to then eat them at home? (*apakah bapak/ibu sering membeli makanan diluar rumah sebagai contoh membeli makanan cepat saji atau membeli diwarung kemudian memakanya dirumah? Seberapa sering?* If yes, where do you usually go, how often, and why? (*Jika iya, dimana bisanya bapak/ibu membeli, seberapa sering, dan mengapa?*)						
**23**	What about packaged foods, for example, instant noodles and snacks like chips, krupuk? (Bagaimana dengan makanan kemasan, sebagai contoh mie instan, snack (camilan), krupuk?						
**24**	What about water, both for drinking and for cooking? Do you buy it or? (*bagaimana dengan konsumsi air bapak/ibu? Apakah menggunakan air yang sama untuk memasak dan minum? Apakah bapak /ibu membeli air tersebut?*						
